# Evaluation of Alternative Sources of Proteins and Other Nutrients with Potential Applications in Fish Nutrition

**DOI:** 10.3390/molecules29102332

**Published:** 2024-05-15

**Authors:** George-Cătălin Muntean, Dorina Simedru, Paul Uiuiu, Claudiu Tanaselia, Oana Cadar, Anca Becze, Aurelia Coroian

**Affiliations:** 1 Department of Fundamental Sciences, University of Agricultural Sciences and Veterinary Medicine Cluj-Napoca, 3-5 Mănăştur Street, RO-400372 Cluj-Napoca, Romania; muntean.gc@gmail.com (G.-C.M.); paul.uiuiu@usamvcluj.ro (P.U.); aurelia.coroian@usamvcluj.ro (A.C.); 2 Research Institute for Analytical Instrumentation Subsidiary, National Institute for Research and Development of Optoelectronics Bucharest INOE 2000, 67 Donath Street, RO-400293 Cluj-Napoca, Romania; dorina.simedru@icia.ro (D.S.); claudiu.tanaselia@icia.ro (C.T.); oana.cadar@icia.ro (O.C.)

**Keywords:** aquaculture, alternative protein, gastropod, plant-based, nutrition, minerals, polycyclic aromatic hydrocarbons

## Abstract

The European Union’s (EU) agricultural self-sufficiency is challenged by its reliance on imported plant proteins, particularly soy from the Americas, contributing to deforestation and greenhouse gas emissions. Addressing the EU’s protein deficit, this study evaluates alternative protein sources for aquaculture, focusing on their nutritional value, elemental content, and polycyclic aromatic hydrocarbons (PAHs). Protein flours from gastropods (*Helix pomatia*, *Arion lusitanicus*, *Arion vulgaris*) and their hepatopancreas, along with plant-based proteins from food industry by-products (oilcakes, coffee grounds, spent brewer’s yeast), were analyzed. Results revealed that snail flour contained the highest protein content at 59.09%, significantly outperforming hepatopancreas flour at 42.26%. Plant-based proteins demonstrated substantial nutritional value, with coffee grounds flour exhibiting a remarkable protein content of 71.8% and spent brewer’s yeast flour at 57.9%. Elemental analysis indicated high levels of essential minerals such as magnesium in hepatopancreas flour (5719.10 mg/kg) and calcium in slug flour (48,640.11 mg/kg). However, cadmium levels in hepatopancreas flour (11.45 mg/kg) necessitate caution due to potential health risks. PAH concentrations were low across all samples, with the highest total PAH content observed in hepatopancreas flour at 0.0353 µg/kg, suggesting minimal risk of PAH-related toxicity. The analysis of plant-based protein sources, particularly oilcakes derived from sunflower, hemp, flax, and pumpkin seeds, revealed that these by-products not only exhibit high protein contents but present a promising avenue for enhancing the nutritional quality of feed. This study underscores the potential of utilizing gastropod and plant-based by-products as sustainable and nutritionally adequate alternatives to conventional feeds in aquaculture, contributing to the EU’s environmental sustainability goals.

## 1. Introduction

The European Union (EU) is largely self-sufficient in agricultural products due to its Common Agricultural Policy (CAP). However, the EU’s livestock sector heavily relies on imported plant proteins, especially soy from Argentina, Brazil, and the USA, for animal feed [1,2]. This reliance links to deforestation and significant greenhouse gas emissions, highlighting the importance of plant proteins in human nutrition. The EU faces a major deficit in plant proteins due to high demand from the livestock sector, leading to heavy dependence on imports, particularly soy.

To address this, the European Union has put forward a number of strategies aimed at mitigating its protein deficit. One such strategy involves encouraging domestic production. This is reflected in the new Common Agricultural Policy (CAP), which supports protein production. Specifically, national strategic plans for the period 2023 to 2027 include provisions for backing legume and protein crops. In addition, the EU is looking at developing alternative sources of proteins that could be utilized for both human and animal nutrition. These alternatives include microbial proteins, algae, and insects, which offer promising avenues for diversification. Furthermore, the EU is focused on enhancing efficiency and sustainability within its agricultural sector. This not only has the potential to reduce the protein deficit but also to improve the overall sustainability of agriculture. Key to this effort is the optimization of resources and the application of digital technologies, which are seen as crucial elements in achieving these goals [1,2,3,4,5,6,7]. In the context of aquaculture, these challenges and strategies become particularly relevant. Fish nutrition is critical in aquaculture, influencing growth, health, and reproduction. Traditionally, fishmeal and fish oil have been the primary protein and lipid sources in aquaculture feeds. However, the sustainability of these resources is a growing concern due to overfishing and ecological impacts. This situation has sparked significant interest in identifying and utilizing alternative, sustainable, and cost-effective protein sources [8,9,10,11,12]. Among the alternatives, plant-based proteins are gaining attention, including options such as soybean meal, canola meal, and corn gluten meal. These sources are appealing due to their sustainability and availability. Nonetheless, they often come with challenges, such as lower protein content and the presence of anti-nutritional factors that can affect digestibility and nutrition [13,14,15]. Insect-based proteins are also emerging as a promising solution. Insects, particularly black soldier fly larvae and mealworms, have been recognized for their high protein content and efficiency in converting low-value organic waste into high-quality protein. This not only addresses the need for sustainable protein sources but also contributes to waste reduction [16,17,18]. Single-cell proteins, encompassing yeast, algae, and bacterial proteins, represent another innovative avenue. These sources are lauded for their high protein content and essential amino acids. The production of single-cell proteins is environmentally sustainable, offering the added benefits of reducing land use and contributing to overall sustainability efforts [19,20,21]. Additionally, by-product proteins derived from the processing of poultry, livestock, and fish are being explored. Utilizing these by-products as protein sources in aquaculture feeds presents a sustainable method to repurpose materials that would otherwise be discarded, further enhancing the environmental sustainability of aquaculture practices. These alternatives highlight the industry’s shift towards more sustainable and environmentally friendly practices, addressing critical concerns over resource sustainability while also striving to meet the nutritional needs of aquaculture operations [22,23,24,25].

Nutrition plays a critical role in aquaculture, mirroring its importance in human health and environmental sustainability. The quest for nutritionally balanced, sustainable feed ingredients is driven by the need to ensure the optimal growth, health, and reproductive success of aquaculture species while minimizing the ecological footprint of production systems [26]. Nutritionally adequate feed is pivotal, not only for the welfare of aquatic species but also for the quality and safety of the final aquaculture products destined for human consumption. As the global population continues to rise, and with it the demand for protein-rich foods, aquaculture stands as a vital component in meeting this demand [27]. Traditionally, fishmeal and fish oil have been primary ingredients in aquaculture feeds; this reliance contributes to overfishing and puts pressure on wild fish populations. Mo and collaborator have reviewed the feasibility of using locally available waste materials, including fish waste, okara, and food waste for producing fish feed. They concluded that food waste is suitable as a component of the diet of farmed fish for species farmed in China, such as tilapia and various Chinese carp [28]. Tran et al. in their systematic review and meta-analysis have quantified the overall effects of various types of insect meal on special growth rate (SGR), feed conversion ratio (FCR), and protein efficiency ratio (PER) of aquatic animals. They concluded that the majority of dietary insect meals had a negative impact on growth performance and that the insect meal must be integrated with other protein sources to obtain high-quality fish feed [29]. However, as Gómez et al. demonstrated in their study, the sustainability of aquaculture feed sources becomes paramount, necessitating a shift towards ingredients that are not only nutritionally complete but also environmentally benign and readily available to ensure the long-term viability of aquaculture as a food source [30]. Integration of these alternative protein sources into fish nutrition presents a viable strategy to address the sustainability challenges in aquaculture, as presented in the study by Li et al. [31]. They have tested five isonitrogenous and isocaloric diets with poultry by-product meal plus soybean meal for juvenile largemouth bass fish feed. The study showed that new nutritional methods are needed to prevent black skin syndrome and liver damage in the largemouth bass that are fed low-fishmeal diets. Using alternative sources of proteins not only reduces the environmental footprint of fish farming but also aligns with the circular economy principles, contributing to waste reduction and resource efficiency as shown in the study by Jiang et al. [32]. Aragao et al. have produced a comprehensive review of the impacts of alternative and novel dietary protein sources, focusing on insect meals, fish gut microbiota and health, stress and immune responses, disease resistance, and antioxidant capacity. The review showed that novel protein sources have functional properties and may exert an overall positive modulatory effect on fish gut microbiota and health but that further research is needed to optimize the nutritional composition of these new types of fish feed [23]. However, the economic implications of this shift, including the cost-effectiveness of alternative protein sources compared to traditional ones, are crucial factors that need consideration [33,34,35].

Fourier Transform Near-Infrared (FT-NIR) spectroscopy is increasingly applied in the rapid analysis of agricultural and food products due to its non-destructive nature and quick turnaround time. However, the application of FT-NIR in analyzing novel feed ingredients presents unique challenges, primarily due to the absence of established standards and the significant matrix effects that can affect the accuracy of the spectral data obtained [36]. To overcome these limitations and enhance the reliability of FT-NIR analysis in novel matrices, calibration correction functions are crucial. These functions are developed by analyzing a subset of samples using both FT-NIR and established chemical methods, allowing the identification and correction of deviations specific to different matrices. This process not only adjusts for inherent matrix variability but also tailors the FT-NIR method to accurately reflect the true chemical composition of novel ingredients. The importance of calibration correction functions lies in their capacity to bridge the gap between rapid spectroscopic techniques and the rigorous, often time-consuming traditional chemical analyses [37]. By integrating calibration correction into the FT-NIR analysis of novel feed ingredients, researchers can utilize this rapid method as a preliminary screening tool while maintaining a level of accuracy that approaches more conventional methods. This dual approach not only streamlines the analytical process but also provides a methodological framework that can be adapted to a wide range of novel materials, ultimately contributing to more efficient and cost-effective feed analysis protocols [38,39].

The incorporation of alternative protein sources in aquaculture feeds, while promising, brings to the fore concerns regarding contaminants such as heavy metals and polycyclic aromatic hydrocarbons (PAHs). Heavy metals, including cadmium, lead, and mercury, can accumulate in aquatic organisms, posing significant health risks to both the aquatic species and humans consuming them. Monge-Ortiz et al. analyzed five heavy metals in experimental diets and fish muscle and concluded that these levels typically show some variations but consistently remain below the risk thresholds [40]. These risks range from neurological damage to increased risk of chronic diseases, underscoring the need for rigorous monitoring and regulation of feed ingredients. Glencross et al. reviewed the available data on risks that need to be considered to enable the production of safe, sustainable, and functional feeds [41]. Due to the disparity of data, they concluded that there is an increasing need for consistency in regulations and standards across the sector, irrespective of international boundaries. Similarly, PAHs, which are formed during the incomplete combustion of organic matter, can accumulate in the food chain. Their presence in aquaculture feeds not only raises concerns over carcinogenic and mutagenic effects but also highlights the broader issue of environmental pollution affecting aquaculture sustainability. Cheng et al. studied different types of food waste as major sources of protein to replace the fish meal used in fish feeds to produce quality fish [42]. The study showed that grass carp and bighead carp fed with food waste feeds were relatively free of PAHs.

Therefore, assessing the risk of heavy metals and PAHs in alternative feed sources is critical, ensuring that the pursuit of sustainability does not compromise the safety and nutritional quality of aquaculture products.

This article aims to comprehensively evaluate and compare various alternative sources of proteins and nutrients, both of animal and plant origin, for their effectiveness in fish nutrition. By doing so, it seeks to address the growing need for sustainable, cost-effective, and nutritionally adequate feed options in aquaculture, filling a crucial research gap. Furthermore, it will explore the potential future directions in sustainable aquaculture practices, including advancements in biotechnology and policy changes, thereby contributing to the ongoing discourse in this field.

## 2. Results

FT-NIR calibration correction factors used for each matrix are presented in Table 1.

### 2.1. Animal Base Protein Sources

**Figure 1:**
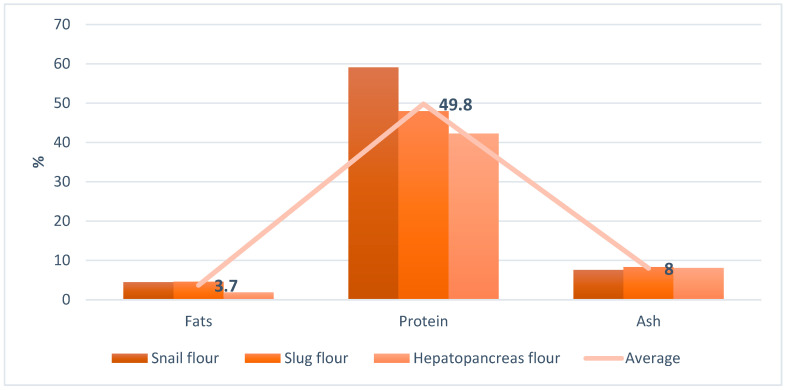
Nutritional characterization of snail, slug, and hepatopancreas protein flour.

**Figure 2:**
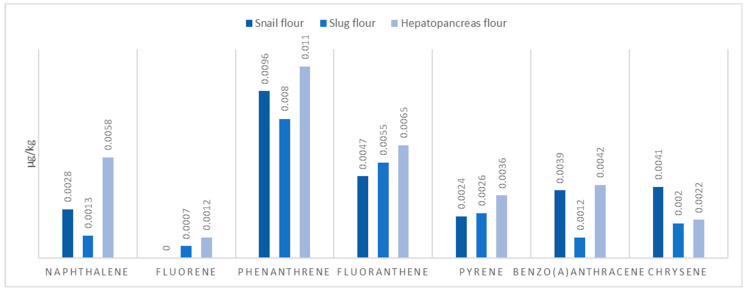
PAH content of protein flours of animal origin.

The results obtained are presented in Table 2 and Figure 1 and Figure 2.

Snail flour showed the highest protein content (59.09%), indicating its superior nutritional value as a protein source, followed by slug flour (47.98%) and hepatopancreas flour (42.26%) (F = 399.736, *p* = 2.1 × 10^−12^). Fats were moderately present in snail (4.5%) and slug flours (4.6%), with hepatopancreas flour having a significantly lower fat content (1.9%, F =798.2531, *p* = 2.5 × 10^−14^), suggesting a leaner protein source. Ash content, indicative of mineral presence, was fairly consistent across the samples, pointing to a good mineral profile in these protein sources (F = 6.92, *p* = 0.00899).

The flours showed a diverse range of elemental contents. Particularly notable were the high levels of magnesium in hepatopancreas flour (5719.10 mg/kg, F = 2,273,554, *p* = 2.87 × 10^−17^) and calcium in slug flour (48,640.11 mg/kg, F = 11,155.91305, *p* = 9.41 × 10^−22^), highlighting their potential as mineral supplements. The levels of potentially toxic elements such as arsenic (As), cadmium (Cd), and lead (Pb) were within acceptable ranges, suggesting these animal-based protein sources are safe for consumption. Hepatopancreas flour had notably higher Cd levels (11.45 mg/kg, F = 970.3446, *p* = 7.08 × 10^−15^), warranting attention.

All flours demonstrated low levels of polycyclic aromatic hydrocarbons (PAHs), with hepatopancreas flour showing the highest total PAH content (0.0353 µg/kg, F = 30,000.99, *p* = 1.52 × 10^−24^). This suggests a relatively low risk of PAH-related toxicity, aligning with safety standards for food products. The statistical significance of the difference in PAH content between the flour types indicates a notable variation, with the hepatopancreas flour containing significantly higher PAHs than the others, yet the levels observed in all flours are still considered low. The animal-based protein flours from snails, slugs, and hepatopancreas exhibit promising nutritional profiles, rich in proteins and minerals, and low in harmful contaminants such as PAHs and heavy metals. These findings, underscored by the significant ANOVA results, support the potential of these flours as innovative and sustainable ingredients in the food and biotechnology sectors, offering new avenues for nutritional applications while adhering to safety and health standards.

### 2.2. Plant-Based Protein Sources

**Figure 3:**
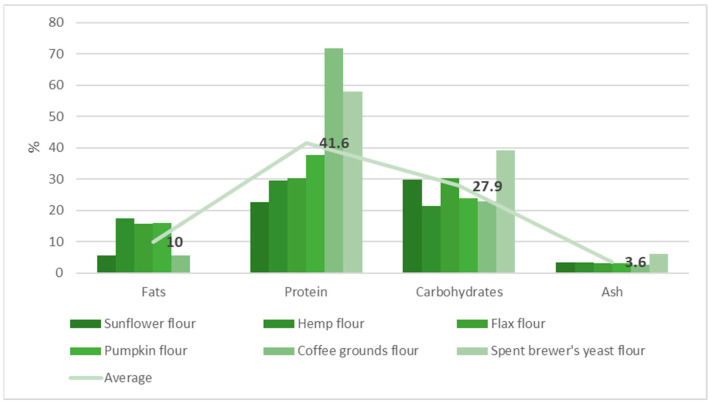
Nutritional characterization of plant-based protein flour.

**Figure 4:**
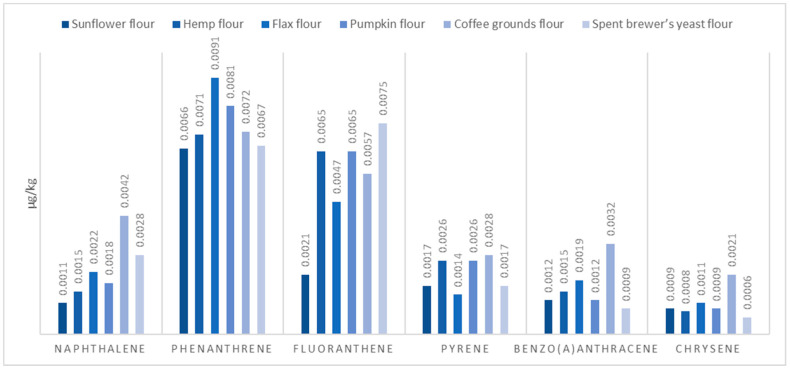
PAH content of protein flours of plant origin.

The results obtained are presented in Table 3 and Figure 3 and Figure 4.

Coffee grounds flour exhibited the highest protein content at 71.8%, followed by spent brewer’s yeast flour at 57.9%, indicating their superior nutritional value as plant-based protein sources (F = 502.01, *p* = 1.86 × 10^−23^). Pumpkin flour also showed a high protein content of 37.6%. Hemp, flax, and pumpkin flours demonstrated high fat content (ranging from 15.7% to 17.5%, F = 67,023.85, *p* = 6.6 × 10^−49^), indicative of their potential as sources of essential fatty acids. Carbohydrate contents varied across the flours, with spent brewer’s yeast flour having the highest at 39.1% (F = 9511.823, *p* = 9.82 × 10^−39^). The ash content, reflecting mineral presence, was highest in spent brewer’s yeast flour (5.98%), suggesting a rich mineral profile (F = 2528.67, *p* = 7.74 × 10^−32^).

The flours showed diverse elemental contents, with pumpkin and hemp flours displaying high levels of magnesium (pumpkin flour: 1940.51 mg/kg; hemp flour: 1858.45 mg/kg, F = 7852.43, *p* = 9.79 × 10^−38^), indicative of their potential as mineral supplements. Potassium levels were notably high across all plant-based flours, especially in coffee grounds flour (7338.9 mg/kg, F = 2148.53, *p* = 5.44 × 10^−31^), which had the highest levels. The levels of potentially toxic elements such as arsenic (As), cadmium (Cd), and lead (Pb) were within acceptable ranges or undetected, suggesting these plant-based protein sources are safe for consumption. It is noteworthy that the cadmium level was detectable but still low in sunflower flour (0.49 mg/kg, F = 46,663.38, *p* = 5.08 × 10^−47^), which may warrant further investigation due to its potential toxicity at higher levels.

All flours demonstrated not significant different levels of polycyclic aromatic hydrocarbons (PAHs), with coffee grounds flour showing the highest total PAH content (0.0252 µg/kg, F = 0.499, *p* = 0.77315). This suggests a relatively low risk of PAH-related toxicity, aligning with safety standards.

The plant-based protein flours exhibit promising nutritional profiles, rich in proteins, essential fatty acids, and minerals, and low in harmful contaminants such as PAHs and heavy metals. These findings support the potential of these flours as sustainable and healthful ingredients in the food industry. The high protein content of coffee grounds and spent brewer’s yeast flours, in particular, highlights their significant potential as novel sources of plant-based protein.

## 3. Discussion

This study presents a comprehensive analysis of protein flours derived from three gastropod species—*Helix pomatia, Arion lusitanicus*, and *Arion vulgaris*—and the hepatopancreas of *Helix pomatia*, focusing on their nutritional value, elemental content, moisture content, and polycyclic aromatic hydrocarbons (PAHs) presence. Our findings reveal significant variations in nutritional composition, elemental concentrations, and PAH levels across these sources, highlighting the potential of these gastropods as alternative protein sources while also underscoring the need for careful consideration of their heavy metal content and PAH contamination. The nutritional analysis indicated that snail flour possesses the highest protein content (59.09%), followed by slug flour and hepatopancreas flour. This aligns with existing research suggesting that gastropods are a high-quality protein source, comparable to traditional livestock proteins [43]. However, the considerably lower protein content in hepatopancreas flour (42.26%) suggests that while it remains a viable protein source, its nutritional value is somewhat inferior to the muscle tissue of snails and slugs. Gomot et al. reported a moisture content of 86.5% for *Helix pomatia*, higher than our observed 82.8%. This discrepancy might be attributed to differences in sample collection or preservation methods prior to analysis. They also found a protein content of 74.5%, surpassing our measurements of 59.09% for *Helix pomatia* and 47.98% for Arion species [44]. The variation in protein content could be explained by factors such as the snails’ diet, the season of collection, or methodological differences in protein quantification. Gomot et al.’s lower fat content of 0.7% compared to our findings suggests a possible influence of environmental conditions or snail age on lipid metabolism. Such seasonal variations could significantly impact nutritional profiles, suggesting a need for standardized collection periods in future research for accurate comparisons. Their higher ash content at 13.6% could indicate a richer mineral diet or a more comprehensive inclusion of shell material in their sample preparation. Çağiltay F. et al. reported humidity level for *Helix aspersa* closely aligned with our findings for *Helix pomatia*, suggesting a consistent water content across these species under similar conditions [45]. The higher protein content in *Helix aspersa* could be indicative of species-specific nutritional profiles or differences in the developmental stages of the snails studied. Their lower fat and ash content relative to our findings could reflect variations in habitat or genetic factors affecting nutrient assimilation and storage. The high fiber content observed in snail and slug flours (over 32%) far exceeds that of conventional protein sources, potentially offering additional health benefits such as improved digestion and liver function [46].

The results demonstrate that calibration correction is a vital step toward validating FT-NIR for use with novel feed ingredients, where standard benchmarks are lacking. This approach aligns with the findings of Li et al. [38], who reported similar improvements in the spectroscopic analysis of blended food products. Unlike conventional methods that rely heavily on direct comparisons to established standards, our methodology accounts for inherent sample variability and adjusts accordingly, thus offering a more reliable analysis for diverse materials.

Elemental analysis revealed substantial differences in the content of essential minerals such as calcium, magnesium, and potassium across the flour types, with snail flour showing particularly high levels of magnesium and calcium. This suggests that snail flour could be an excellent dietary source of these crucial minerals, supporting bone health and metabolic functions [47]. However, the high concentrations of heavy metals such as cadmium in hepatopancreas flour raise significant health concerns, given cadmium’s association with kidney damage and skeletal disorders [48]. The cadmium and lead levels analyzed by Corda et al. reveal a broad range that includes values both above and below our findings [49]. The wide variance in heavy metal content highlights the impact of environmental pollution on snail habitats, with soil and water contamination being likely contributors. While we identified high protein and essential mineral contents in gastropod flours, the presence of anti-nutrients remains a critical concern for their application in aquaculture feeds. For instance, gastropods are known to accumulate oxalates and phytates, which could impair mineral absorption in fish, posing a risk to their health and growth. Further research should, therefore, prioritize the quantification of these anti-nutrients in gastropod flours to assess fully their suitability as feed ingredients [50].

Our study’s lower contaminant levels may reflect the selection of cleaner collection sites or differences in bioaccumulation capacities among snail species. The detection of PAHs in all flour types, albeit at low concentrations, warrants attention due to the carcinogenic and mutagenic properties of these compounds [51]. The relatively higher total PAH concentration in hepatopancreas flour compared to snail and slug flours may reflect differences in the bioaccumulation capacities of these tissues, highlighting the importance of monitoring environmental contaminants in gastropod-derived food products. The observed variability in nutritional and elemental compositions, as well as the PAH levels, underscores the need for standardized production and processing protocols to ensure the safety and nutritional quality of gastropod-based food products. Furthermore, our study opens avenues for future research into the health implications of long-term consumption of these alternative protein sources, particularly concerning heavy metal accumulation and exposure to PAHs. In conclusion, the protein flours derived from *Helix pomatia, Arion* spp., and the hepatopancreas of *Helix pomatia* exhibit promising nutritional profiles, rich in proteins and essential minerals, making them potential sustainable alternatives to traditional animal protein sources. However, concerns regarding heavy metal content and PAH contamination highlight the need for comprehensive risk assessments and the development of guidelines to maximize the health benefits of consuming these novel protein sources.

This study faced several constraints that warrant consideration. The reliance on rainfall for sample collection introduced variability in sample sizes and collection times, limiting the standardization across experimental units. Only a limited number of batches were analyzed (3 to 8 events per ingredient), which may affect the generalizability of our findings.

The innovative valorization of by-products from the food industry, such as oilcakes, coffee grounds, and spent brewer’s yeast, into plant-based protein flours, addresses the dual challenges of environmental sustainability and the increasing global protein demand. Particularly, coffee grounds flour and spent brewer’s yeast flour exhibited significant protein contents of 71.8% and 57.9% DM, respectively, highlighting their potential to serve as high-quality protein sources. These findings resonate with the growing body of literature that supports the circular economy model by converting waste into valuable food ingredients [52]. Our analysis revealed notable variations in the nutritional profiles of the studied plant-based protein flours. For instance, hemp flour demonstrated a high fat content of 17.5% DM, suggesting its potential as a valuable source of essential fatty acids. Meanwhile, pumpkin flour was identified as the most protein-rich among the seed oilcakes, with a protein content of 37.6% DM. The results are similar to that of Petraru et al.’s study with proteins ranging from 20.15% to 21.60%, and the remaining oil from 15.77% to 14.16% for sunflower oilcakes [53]. Sinkovič and Kolmanič also found similar levels in the study on pumpkin oilcakes regarding crude fats content 8.7–10.3%, while the reported protein level was somewhat higher at 65.3–68.9% [54]. This diversity in nutritional composition underscores the potential of these flours to cater to a range of dietary needs and preferences, aligning with findings from Moura et. al. that emphasize the importance of diversifying plant-based protein sources [55].

**Figure 5:**
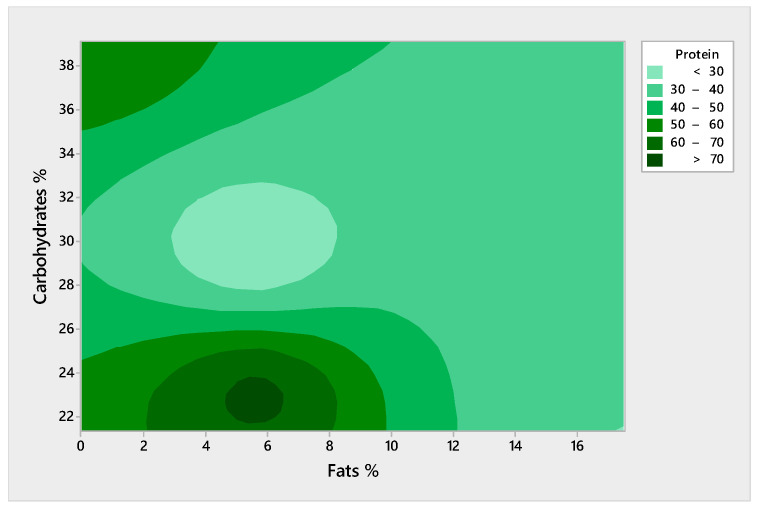
Contour plot of proteins versus carbohydrates and fats for plant-based proteins.

Figure 5 offers a contour plot analysis illustrating the intricate balance between carbohydrates, fats, and protein contents in plant-based proteins. The fat percentage is plotted on the *x*-axis, scaling from 0% to over 15%, and the carbohydrate percentage is on the *y*-axis, ranging approximately from 22% to 38%. The protein content, delineated by the green-shaded contours, is presented as percentages increasing from less than 30% to over 70%. The darkest green area indicates the highest protein content (above 70%), which is concentrated around the lower fat percentages (between approximately 2% to 4%) and mid-range carbohydrate percentages (around 24% to 26%). This suggests an optimal region for plant-based proteins that could provide a balanced macronutrient profile suitable for aquaculture nutrition, especially when aiming for high-protein formulation with moderate energy contributions from fats and carbohydrates. The lighter green regions radiating outward correspond to decreasing protein percentages, showing that an increase in fat percentage is generally associated with a reduction in protein concentration. This pattern demonstrates the inverse relationship between fat content and protein concentration in plant-based protein sources. Notably, the data suggest that carbohydrate percentages have a less pronounced impact on protein levels compared to fats within the studied range.

The elemental content analysis revealed that pumpkin flour contained the highest magnesium concentration (1940.51 mg/kg), highlighting its nutritional value. Moreover, the low levels of potentially harmful elements and PAHs across all samples, with coffee grounds flour showing a total PAH content of only 0.0252 µg/kg, demonstrate the safety of these protein sources for human consumption. These results align with the safety standards established by regulatory authorities and are consistent with the findings of Lee et al. (2019) [56], who reported the importance of monitoring elemental and PAH contents in food products. By demonstrating the feasibility of transforming food industry by-products into nutrient-rich protein flours, this study contributes to the development of more sustainable food systems. The approach not only mitigates waste but also reduces the environmental footprint associated with traditional protein production methods [57]. Further research into the life cycle assessments (LCAs) of these protein flours could offer deeper insights into their environmental impacts, reinforcing the findings of this study. Although our study did not directly analyze plant secondary metabolites, their potential impact on animal health cannot be overlooked. Compounds such as saponins, present in various plant sources, have been shown to exhibit both growth-promoting and toxic effects, depending on the dosage [58].

## 4. Materials and Methods

### 4.1. Animal-Based Protein Sources

In this study, we analyzed protein flours from 4 sources: *Helix pomatia, Arion* spp., and hepatopancreas from *Helix pomatia* that had resulted from the production of snail meat in a production unit.

*Helix pomatia* and *Arion* spp. were manually collected from Cluj County, Romania. The collection took place from April to September 2023. Given the dependence on rainfall for collection, a flexible collection protocol was established. Each collection event was treated as a separate batch, and samples from individual events were processed independently. There were five collection events for *Helix pomatia*, eight for *Arion* spp., and the hepatopancreas was collected three times from the processing facility.

After manual collection, *Helix pomatia* specimens were immediately immersed in a euthanasia solution composed of 50% food-grade alcohol (96% ethanol) and 50% distilled water, ensuring ethical euthanasia in accordance with animal welfare standards. Subsequently, at laboratory facilities, the specimens were transferred to cold water to remove any surface impurities, followed by a meticulous rinsing process. The snails were then manually extracted from their shells, with the muscular tissues (snail meat) being separated from the internal organs. This separation was executed with precision to maintain the integrity of the muscle tissue intended for subsequent analyses. The obtained muscle tissue was preserved at a temperature of 4 °C under refrigeration for up to 24 h. For long-term preservation, the tissue was frozen, with the freezing process tailored according to the time interval until further processing of the tissue into snail flour.

*Arion* spp. specimens were processed in the same manner, but the shell removal step was omitted as it was not necessary for these species.

The processing of muscle tissues from *Helix pomatia* and *Arion* spp. species was standardized to ensure the consistency and quality of the final product. This procedure included the following steps:

- Drying: Initially, the muscle tissue underwent a dehydration process in a drying oven (UFE 400 oven from Memmert, Büchenbach, Germany). This process was conducted at a controlled temperature of 60 °C. The aim of this stage was to reduce the moisture content in the tissue until a constant mass was achieved, thus ensuring complete and uniform drying of the biological material.
- Mass Monitoring: The mass of the tissue was periodically monitored (WSP4000/C/2, Partner Radwag, Radom, Poland) to determine the point of constant mass achievement, indicating the completion of the dehydration process. This stage is crucial for ensuring the quality and uniformity of the final product.
- Grinding: After dehydration completion, the dried tissue was transferred for grinding. The grinding process was performed using specialized equipment to achieve a fine and uniform granulation of the material (PM 100, Retsch GmbH, Haan, Germany). The purpose of this stage was to transform the dehydrated tissue into a powder form, thereby facilitating its subsequent use in various applications, including protein extraction.

The hepatopancreas from *Helix pomatia* was harvested through a specialized process in a snail meat production unit, beginning with the initial preparation where live snails were placed in a heat-resistant plastic container and covered with boiling water. This was followed by a thermal treatment, keeping the snails in hot water for 3–4 min until the meat near the shell entrance turned yellowish, indicating proper pre-cooking. After draining the water, the snail was carefully removed from the shell using a small two-pronged metal fork. The hepatopancreas was then meticulously separated from the rest of the snail meat using sharp scissors. For preservation, the obtained hepatopancreas was refrigerated at 4 °C for up to 24 h, and for longer storage, it was frozen, with the duration determined based on the planned processing time into hepatopancreas flour.

The production of flour from the hepatopancreas of *Helix pomatia* involved a series of essential steps. The muscle tissue was first subjected to dehydration in a drying oven at a controlled temperature of 85 °C to adequately reduce the microbiological load and the moisture content until a constant mass was achieved, ensuring complete and uniform drying of the biological material. The mass of the tissue was then periodically monitored to ascertain the achievement of a constant mass, signaling the end of the dehydration process, a crucial step for ensuring the quality and uniformity of the final product. Following dehydration, the dried tissue was transferred for grinding, where it was processed using specialized equipment to achieve fine and uniform granulation.

### 4.2. Plant-Based Protein Sources

**Figure 6:**
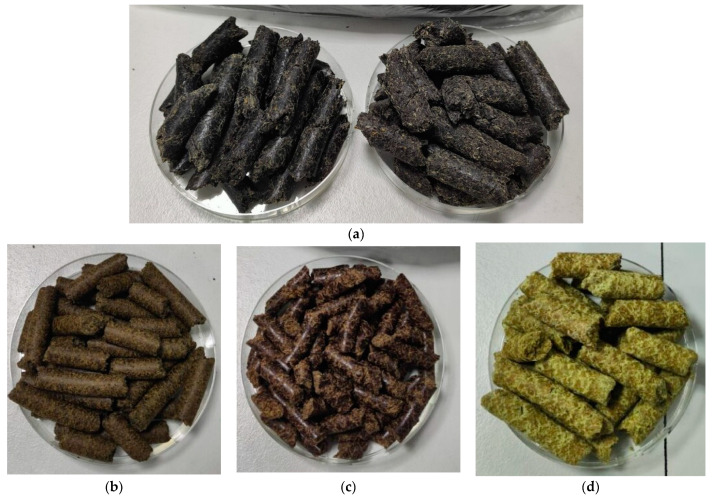
Different oilcakes used in the study: (**a**) sunflower seed oilcake; (**b**) hemp seed oilcake; (**c**) flax seed oilcake; (**d**) pumpkin seed oilcake.

Plant-based protein flours were obtained from by-products of the food industry. These included different oilcakes such as sunflower, hemp, flax, and pumpkin seed cakes, coffee grounds, and spent brewer’s yeast. All these sources were obtained from Cluj County producers of cold-pressed vegetable oils, coffee shops, and small-batch beer producers. The samples were collected from April to October 2023. There were nine sunflower oilcake samples, five hemp oilcake samples, three flax oilcake samples, three pumpkin seed oilcake samples, as well as twelve coffee grounds samples and seven spent brewer’s yeast samples. The process of obtaining flour from oilcakes involved cleaning and sorting the oilcakes to remove impurities and unextracted seed residues, ensuring the uniformity and purity of the base material. The oilcakes were then ground using specialized equipment, allowing for granularity adjustment to meet the final product’s specific requirements. After grinding, the flour underwent sifting and classification to separate particles of different sizes, ensuring uniform granulation and contributing to the consistency of the final product (Figure 6).

In the study, the process of obtaining flour from coffee grounds involved several key steps. Initially, the coffee grounds were collected and subjected to a cleaning process to remove impurities and liquid residues, a measure critical for ensuring the purity and quality of the flour. Following the cleaning, the wet grounds were dried in ovens set at a temperature of 60 °C. This drying step was carefully managed to reduce the moisture content while preserving the aromatic compounds inherent in the coffee grounds. Once the drying phase was completed, the grounds were finely ground. The final step in the process involved sifting the resulting flour to remove any large particles.

In the conducted study, spent yeast was collected from the bottom of fermentation vessels after the brewing process. The collected yeast was then subjected to a cleaning process to remove impurities and beer residues, which involved separating liquids and other residual substances. Following the cleaning, the yeast underwent a concentration process through centrifugation (EBA21, Hettich, Kirchlengern, Germany), aimed at reducing its water content. Subsequently, the concentrated yeast was dried in ovens at a temperature of 60 °C, a step taken to further reduce its moisture content. Once dried, the yeast was ground to produce flour. In the final stage of the process, the resulting flour was sifted to eliminate large particles, thereby ensuring a fine and uniform texture of the final product.

### 4.3. Reagents and Materials

All solvents were HPLC grade from VWR (Darmstadt, Germany), with ultra-pure water obtained using the ULTRACLEAR UV UF EVOQUA Purification system (Pittsburgh, PA, USA) and Florisil 200 mesh, fine powder from Supelco (St. Louis, MO, USA).

### 4.4. Nutritional Value Analysis

The measurements were carried out on the Tango Bruker (Billerica, MA, USA) using a modified method from Masithoh et al. [59]. Flour samples were measured directly, without extraction. The method parameters were 92 s of measurement time, 16 cm^−1^ resolution, and a rotating scan type. The results were compared to the calibration curves supplied by Bruker. The results are expressed as a percentage.

Acknowledging the typical matrix-dependency of FT-NIR and the absence of established standards for novel feed ingredients, we calculated a calibration correction parameter tailored to our novel ingredients by comparing a subset of our samples analyzed with both FT-NIR and standard chemical methods for proteins (Kjeldahl) and lipids (Soxhlet extraction). A modified version of Mæhre et al.’s methods was used for Kjeldahl determination. A quantity of 1 g of the homogenized sample was hydrolyzed with concentrated sulfuric acid and copper catalysts, followed by neutralization and titration. The total nitrogen content obtained was converted to protein content using a traditional conversion factor of 6.25 [60]. For the lipid determination, a method adapted from Manirakiza et al. was used. A quantity of 5 g of the oven-dried and ground sample was placed in a filter paper thimble. The thimble was then loaded into the Soxhlet extractor, and hexane was used as the solvent. The extraction process continued for 6 h, allowing the solvent to repeatedly percolate through the sample and dissolve the lipids. After extraction, the solvent was removed using a rotary evaporator, and the residue was dried to a constant weight to determine the total lipid content [61].

For each set of ingredients grouped by similar matrix characteristics, the first two samples were chosen based on their compositional differences, which included variations in protein and lipid contents. This methodical selection ensures that the calibration correction function would be robust across different ingredient types and representative of the broader dataset. The calibration correction parameter was calculated through a systematic comparison of the FT-NIR results with those obtained from the standard chemical methods. The datasets obtained from both FT-NIR and standard chemical analyses underwent statistical analysis in order to identify any systematic biases or deviations that could affect the accuracy of the spectroscopic data. Following this, using regression analysis, a calibration correction parameter was calculated for each type of matrix encountered in the sample set. This parameter was subsequently applied to the FT-NIR Tango software (version 8.5) to adjust the results, ensuring they closely matched those obtained from the established chemical methods. This integrated approach provides a robust framework for enhancing the reliability of FT-NIR in the analysis of novel materials where established standards are not available.

### 4.5. Humidity Content Analysis

Moisture content was determined in accordance with SR ISO 1442:2023 [62]. The principle of the method involved drying samples from the test specimens at a temperature of 103 °C until a constant mass was achieved (UFE 400 oven from Memmert, Büchenbach, Germany). The difference in mass before and after the drying process of the sample provided a measure of the water content. The water content was then expressed as a percentage of mass.

### 4.6. Elemental Analysis

The determination of elemental concentrations was conducted using a modified version of the Karasakal method [63] with an Inductively Coupled Plasma Optical Emission Spectrometer (ICP-OES), specifically the Optima 5300 DV Perkin Elmer model, following microwave-assisted digestion of the samples. A quantity of 500 mg of each sample was subjected to microwave digestion using 8 mL of 65% HNO_3_ and 2 mL of 30% H_2_O_2_. The digestion program used is detailed in Table 1. After cooling to room temperature, each sample was diluted to 25 mL with ultrapure water and then filtered through a 0.45 μm cellulose membrane filter. Blank samples were prepared in a similar manner. The concentrations of trace elements in the mineralized solutions were determined using Inductively Coupled Plasma Mass Spectrometry (ICP-MS).

### 4.7. PAH Analysis

The methodology employed was a modified version of a previously developed method for analyzing various food samples.

A 10 g sample was homogenized using a laboratory mixer (Oster, Model 6808-051, McMinnville, TN, USA). To this homogenate, 50 mL of 0.4 M KOH solution in ethanol and water (in a 9:1 ratio) was added for saponification. This process was carried out in an ultrasonic bath (SONOREX, Bandelin, RK 103H, Berlin, Germany) for 30 min at 60 °C. Following saponification, the mixture was filtered through filter paper (Whatman, 110 mm, 1.6 μm, Maidstone, UK). The filtrate was then extracted twice, with 15 mL of cyclohexane each time. The supernatant obtained was further purified using a Florisil column. Subsequently, the purified extract was evaporated (RapidVap Vertex Dry Evaporator, Labconco, Kansas City, MO, USA) to dryness under a stream of nitrogen and finally reconstituted in 1 mL of acetonitrile. Before injection for analysis, the samples were further filtered through 0.45 µm cartridges.

Analysis was performed using a Perkin Elmer (Waltham, MA, USA) 200 Series High-Performance Liquid Chromatograph (HPLC) equipped with a fluorescence detector (FLD). This setup facilitated the separation of 15 polycyclic aromatic hydrocarbons (PAHs), including naphthalene, acenaphthene, fluorene, phenanthrene, anthracene, fluoranthene, pyrene, benzo(a)anthracene, chrysene, benzo(b)fluoranthene, benzo(k)fluoranthene, benzo(a)pyrene, dibenzo(a,h)anthracene, benzo(g,h,i)perylene, and indeno(1,2,3-cd)pyrene. The separation process was carried out with an Inertsil ODS-P 5 µm, 4.6 × 150 mm column, which was maintained at a temperature of 24 °C. An injection volume of 50 µL was used for the analysis. The mobile phase comprised a gradient mixture of water and acetonitrile, and a time-programed FLD detector was utilized for detecting the PAHs. The content of PAHs in the samples was quantified and expressed in nanograms per gram (ng/g).

### 4.8. Statistical Evaluation

The study employed the Analysis of Variance (ANOVA) method to analyze the data, with results presented as mean values along with their standard deviation (SD). For all statistical analyses, the Minitab software, specifically version 17.0 for Windows (Minitab LLC, State College, PA, USA), was used.

## 5. Conclusions

This study highlights the feasibility of using gastropod and plant-based protein flours as sustainable, nutritional alternatives to conventional animal feed in aquaculture. Gastropod-derived flours offer high-quality protein and fiber, although attention to heavy metal and PAH contamination is crucial. Plant-based flours, especially those from coffee grounds and spent brewer’s yeast, present a highly sustainable and nutritionally rich option, supporting the EU’s drive towards self-sufficiency and environmental sustainability in protein production. Future directions should focus on optimizing production processes, ensuring safety, and assessing the economic viability of these alternative sources in aquaculture practices. The findings support a broader adoption of circular economy principles in the aquaculture sector, contributing to the global efforts in sustainable food production and environmental conservation.

## Figures and Tables

**Table 1:** FT-NIR calibration correction factors.

Sample Matrix	Protein	Fats
Snail Flour	1.024	1.037
Slug Flour	1.055	1.021
Hepatopancreas Flour	1.077	1.023
Sunflower Flour	1.034	1.014
Hemp Flour	1.051	1.025
Flax Flour	1.027	1.020
Pumpkin Flour	1.020	1.011
Coffee Grounds Flour	1.029	1.009
Spent Brewer’s Yeast Flour	1.014	ND

**Table 2:** The total elemental content of protein flours of animal origin.

Compound Name	UM	Snail Flour		Slug Flour		Hepatopancreas Flour	
		Average	SD	Average	SD	Average	SD
Na	mg/kg	177.12	11.57	5549.02	95.71	2470.85	85.06
Mg	mg/kg	3821.48	82.57	2631.10	61.54	5719.10	101.24
Al	mg/kg	21.78	2.71	211.90	15.33	62.50	5.91
Si	mg/kg	285.01	5.24	271.10	11.09	115.65	9.54
K	mg/kg	15,831.76	345.12	16,688.02	651.02	18,623.77	751.30
Ca	mg/kg	8409.02	235.13	48,640.11	851.24	14,770.03	245.13
Mn	mg/kg	15.58	0.99	283.25	19.04	96.80	4.98
Fe	mg/kg	96.18	8.24	263.05	18.73	212.10	18.37
Ni	mg/kg	4.18	0.42	1.55	0.09	1.21	0.59
Cu	mg/kg	28.77	1.84	18.75	2.03	129.70	11.43
Zn	mg/kg	43.12	3.51	112.95	8.42	116.65	8.82
As	mg/kg	<LQ	ND	1.56	0.74	0.11	0.02
Se	mg/kg	0.22	0.01	<LQ	ND	0.34	0.05
Sr	mg/kg	8.30	0.76	109.20	7.13	88.10	2.46
Cd	mg/kg	0.06	0.001	1.17	0.85	11.45	0.93
Ba	mg/kg	1.60	0.09	32.35	2.67	98.80	6.44
Pb	mg/kg	0.24	0.02	0.53	0.0002	1.72	0.0003

LQ—quantification limit, 0.005 mg/kg; ND—not determined.

**Table 3:** The total elemental content of protein flours of plant origin.

Analysis	UM	Sunflower Flour		Hemp Flour		Flax Flour		Pumpkin Flour		Coffee Grounds Flour		Spent Brewer’s Yeast Flour	
		Average	SD	Average	SD	Average	SD	Average	SD	Average	SD	Average	SD
Na	mg/kg	96.25	6.41	110.6	9.41	354.55	26.55	127.71	10.02	33.35	3.45	5.5	0.41
Mg	mg/kg	1245.8	95.03	1858.45	102.78	1407.25	76.95	1940.51	143.67	936.71	40.12	90.9	7.84
Al	mg/kg	3.03	0.05	5.05	0.39	3.54	0.22	15.2	1.06	0.16	0.01	6.1	0.51
Si	mg/kg	<LQ	ND	29.3	1.99	<LQ	ND	<LQ	ND	58.4	4.61	5.9	0.42
K	mg/kg	3431.75	261.09	3843.35	70.03	3484.25	251.08	3951.55	297.78	7338.9	120.34	417.2	38.47
Ca	mg/kg	1045.35	52.61	761.12	39.13	1449.65	307.21	153.02	84.31	221.15	25.04	179.9	12.57
Mn	mg/kg	16.71	1.05	94.45	7.43	26.22	2.09	29.51	2.38	<LQ	ND	0.39	0.01
Fe	mg/kg	28.75	2.01	91.51	7.68	49.45	3.84	87.45	6.26	4.10	0.37	2.54	0.14
Ni	mg/kg	6.11	0.22	5.95	0.14	1.50	0.08	1.67	0.09	0.08	0.01	0.12	0.01
Cu	mg/kg	14.65	1.21	12.61	0.91	12.51	1.05	8.45	0.69	3.46	0.31	0.81	0.01
Zn	mg/kg	31.45	3.04	41.15	3.82	43.85	3.61	43.25	3.23	1.74	0.09	1.16	0.09
As	mg/kg	<LQ	ND	<LQ	ND	<LQ	ND	<LQ	ND	<LQ	ND	<LQ	ND
Se	mg/kg	<LQ	ND	<LQ	ND	<LQ	ND	<LQ	ND	<LQ	ND	<LQ	ND
Sr	mg/kg	3.74	0.18	5.72	0.24	8.82	0.94	2.85	0.11	0.92	0.05	0.19	0.01
Cd	mg/kg	0.49	0.01	<LQ	ND	0.28	0.02	<LQ	ND	<LQ	ND	0.01	0.001
Ba	mg/kg	1.64	0.43	2.92	0.13	1.31	0.04	1.14	0.09	<LQ	ND	0.04	0.002
Pb	mg/kg	0.15	0.01	0.14	0.01	0.11	0.01	0.16	0.01	<LQ	ND	0.01	0.001

LQ—quantification limit, 0.05 mg/kg. ND—not determined.

## Data Availability

The data presented in this study are available upon request from the corresponding author. The data are not publicly available due to privacy restrictions.
